# Leaders in collective migration: are front cells really endowed with a particular set of skills?

**DOI:** 10.12688/f1000research.11889.1

**Published:** 2017-10-27

**Authors:** Eric Theveneau, Claudia Linker

**Affiliations:** 1 Centre de Biologie du Développement (CBD), Centre de Biologie Intégrative (CBI), Université de Toulouse, CNRS, UPS, France; 2 Randall Division of Cell & Molecular Biophysics, King's College London, London, UK

**Keywords:** collective cell migration, migratory cells, cell topology

## Abstract

Collective cell migration is the coordinated movement emerging from the interaction of at least two cells. In multicellular organisms, collective cell migration is ubiquitous. During development, embryonic cells often travel in numbers, whereas in adults, epithelial cells close wounds collectively. There is often a division of labour and two categories of cells have been proposed: leaders and followers. These two terms imply that followers are subordinated to leaders whose proposed broad range of actions significantly biases the direction of the group of cells towards a specific target. These two terms are also tied to topology. Leaders are at the front while followers are located behind them. Here, we review recent work on some of the main experimental models for collective cell migration, concluding that leader-follower terminology may not be the most appropriate. It appears that not all collectively migrating groups are driven by cells located at the front. Moreover, the qualities that define leaders (pathfinding, traction forces and matrix remodelling) are not specific to front cells. These observations indicate that the terms leaders and followers are not suited to every case. We think that it would be more accurate to dissociate the function of a cell from its position in the group. The position of cells can be precisely defined with respect to the direction of movement by purely topological terms such as “front” or “rear” cells. In addition, we propose the more ample and strictly functional definition of “steering cells” which are able to determine the directionality of movement for the entire group. In this context, a leader cell represents only a specific case in which a steering cell is positioned at the front of the group.

## Introduction

Collective cell migration is the coordinated motion of a group of cells that emerges from their chemical, physical and/or mechanical interaction ^1– 3^. Collective cell migration is a prevalent feature during development. Morphogenesis is achieved by orchestrating cell specification and patterning and coordinating movement of cells over time. The main movements of cells are intercalation and single or collective cell migration. Collectively migrating groups can be formed of epithelial or mesenchymal cells originating from one cell population or composed of a mixture of cell types. Different modes of cooperation have been described, from the tightly interconnected epithelial sheets or strands to the loose streams of individual cells communicating via transient contacts and secreted signals.

Often the role of the different cells in the collectively migrating group is described through division of labour in two categories: leader or follower cells ^4– 6^. Leader cells are located at the front of the group and allegedly control directionality of movement. Khalil and Friedl originally proposed that leaders would act by “exploring the environment, finding the path, generating traction force and proteolytically remodelling the extracellular matrix” ^6^. Accordingly, follower cells trail leaders and have been proposed to be defined by their inability to generate their own tracks ^7^. Since this original framing of the concept, experimental data from epithelial and mesenchymal models of collective migration, which we comment on hereafter, indicate that (i) directionality of the group is not systematically set by cells located at the front, (ii) generation of traction forces and protrusive and proteolytic activities are not specific to front cells, and (iii) protrusive activity and the ability to guide a group of cells are uncoupled.

It was recently recognized that, in conjunction with chemical signals, the physical interaction between cells plays a fundamental role during collective migration ^2^. Therefore, unveiling the mechanisms regulating this process will require an interdisciplinary approach comprising biology, physics and mathematics. Multiple agent-based or continuous mathematical models of collective cell movement have been proposed and reviewed elsewhere ^8– 17^. These models incorporate strategies previously used in a variety of systems from swarming of bacteria, flocking of birds or schooling fish to foam dynamics. It turns out that modulation of cell motility and cell mechanical interactions are the key parameters sufficient to recapitulate the main cell behaviours observed *in vivo* and *in vitro*. Interestingly, in the context of mechanically interacting cells, a small fraction of cells responding to an external signal is sufficient to significantly bias the direction of movement ^10^. Importantly, though, these cells do not need to be at the front of the population or to be organized into a specific subgroup. They can act while being evenly scattered across the cell population. Additionally, models of epithelial cell monolayers in which no differentiation into leader cells is implemented ^18^ still recapitulate experimental data on wound closure ^19^. Furthermore, models of cooperation among mesenchymal cells show that local cell interactions and confinement, in the absence of leader-follower identities and external gradients, are sufficient to achieve directional movement ^9^. Overall, *in silico* studies suggest that (i) directional collective migration can occur in the absence of a specialized subset of cells and (ii) when specialized cells are present, these do not need to be localized at the front of the group to drive collective migration.

Altogether, experimental and *in silico* data indicate that the original do-it-all leader and passive follower nomenclature is not suitable. In addition, we think that the leader-follower terminology is biased as it combines function and position when these may be uncoupled and leads to the expectation that front cells have a prominent guidance role. Therefore, we propose to introduce a purely functional “steering cell” term, of which a looser leader cell definition might represent a specific subtype of specialized front cell. The steering cell term should, in turn, be used together with purely topological terms such as “front cell” or “rear cell”.

## Epithelial models of collective cell migration

There are several models of collective migration of epithelial cells. Some of the most common include mammalian epithelial monolayers in culture ^20^, sprouting blood vessels ^21^, tracheal cells ^22^ and germ-band extension ^23^ of the *Drosophila* embryo as well as border cells of the drosophila egg chamber ^24^.

Epithelial monolayers are extremely large systems formed by hundreds of cells. Experimental setups start with a confluent monolayer in which space is generated by wounding, scratching or lifting barriers that separate two populations. A limited number of leader cells emerge at the free edge. It has been shown that Notch lateral inhibition, mechanical cues and topology ^25, 26^ are pivotal for leader cell selection. Indeed, once leaders emerge, they take on a specific morphology characterized by a pseudomesenchymal phenotype with a large lamellipodia at the free edge ^20, 25, 27, 28^. Leader cells are linked to the rest of the group by actomyosin cables ^29^. Such cables mechanically couple leaders with their immediate neighbours and play a role in preventing adjacent cells from displaying protrusive activity. Follower cells, located behind leaders, establish their directionality through communication with leaders. This relies on signalling molecules and on the local balance of forces. In extreme cases, leader cells can pull a so-called finger, formed of multiple follower cells, from the epithelial monolayer to invade the free space ^30, 31^. The whole structure has actin cables running along the side membranes from the leader at the tip through the several rows of followers behind. Thus, the finger behaves as a super cell with a distribution of actin polymerization and actomyosin contraction spanning several cells along the finger ^30^. In this situation, motility is not restricted to front cells, even if leaders can exert more force ^28, 29, 32, 33^. The number of leader cells has been shown to be regulated by Notch lateral inhibition ^25, 26^ and mechanical coupling that prevent follower cells from becoming leaders ^25, 26^. Interestingly, during wound healing, metalloproteinase expression differs between leading and trailing populations (reviewed in 34– 36). Matrix metalloproteinase 1 (MMP1), 9 and 10 are expressed in keratinocytes at the leading edge ^35^, while other MMPs are expressed by keratinocytes away from the leading edge, such as MMP3 ^36^ and MMP28 ^37^, or by stromal cells near the wound, like MMP8 ^38^. In summary, in epithelial sheets, specialized front cells emerge at the leading edge and are maintained for long periods of time through local interactions among migratory cells, but matrix remodelling and traction forces are not restricted to these specialized cells.

In invasive carcinoma (tumour of an epithelial tissue), cancer cells invade by themselves with tip cells either adopting a pseudomesenchymal phenotype or associated with non-tumoral cells such as cancer-associated fibroblasts (CAFs) ^39^. In the former case, tip cells are similar to leader cells emerging in the aforementioned epithelial monolayers. The emergence of leader cancer cells can be enhanced via environmental changes such as local increase of compression ^40^. In the latter case, CAFs are thought to be induced from various cell types to become invasive, capable of matrix remodelling (that is, expressing MMP2, 9 and 14) and able to determine the directionality of the metastatic group. CAFs can generate tracks in the matrix for cancer cells to use ^41, 42^ and even directly pull cancer cells out of a tumour ^43^. The strand formed by a leading CAF followed by carcinoma cells resembles the structure of epithelial fingers described above.

**Figure 1.**
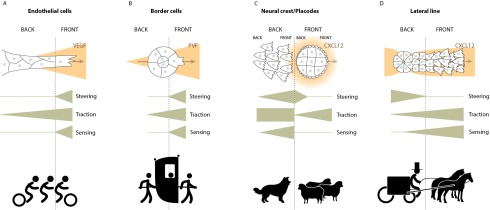
The many ways of steering a cell collective. ( **A**) Endothelial cells. One tip cell responds to vascular endothelium-derived growth factor (VEGF) and adopts a pseudomesenchymal phenotype with distinctive lamellipodia. This works as a bicycle with multiple riders (tandem, triplets, quads or quints). The front cyclist (the tip cell) is responsible for sensing and steering while traction force is shared among cyclists. ( **B**) Border cells. One cell responds better to platelet-derived/vascular endothelium-derived growth factor homologue (PVF) and adopts a pseudomesenchymal phenotype with a distinctive protrusion. This works as several persons carrying a sedan chair. The front person (front cell) is responsible for sensing and steering and exerts traction force but all cells are mechanically coupled and traction is shared. ( **C**) Placodes and neural crest cells. Epithelial placodes and mesenchymal neural crest cells have intrinsic motility (low for placodes and high for neural crest cells) but no directionality on their own. Neural crest cells sense placodes via Cxcl12. Placodes do not sense an external cue but are repelled by repeated physical contact with the neural crest. This works as a sheepdog (neural crest cells) and livestock (placodes) interaction. The sheepdog (highly motile) is attracted by the livestock (gregarious). The livestock only moves to go away from the sheepdog. When they are separated, motility is conserved but directionality is lost. ( **D**) Lateral line primordium. Front cells are mesenchymal while back cells are epithelial. Homogenous Cxcl12 distribution is transiently and locally converted into a gradient by the back cells. Front cells sense this gradient. This works as horse-drawn carriage. The driver in the carriage (back cells) is responsible for steering while horses (front cells) follow available instructions and pull the whole structure. When the two are separated, horses remain motile but lose directionality while the carriage is immobile.

Angiogenesis is the process by which new blood vessels are formed from existing vessels. At the onset of vessel sprouting, all endothelial cells are able to respond to the chemoattractant VEGF (vascular endothelium-derived growth factor) by upregulating the expression of the Notch ligand Dll4 (Delta like-4), which results in the downregulation of VEGF receptor in adjacent cells. In this manner, the VEGF-Dll4/Notch lateral inhibition pathway generates heterogeneity in the population and singles out tip (leader) cells ^44^. Importantly, high levels of VEGF activity also determine tip cell morphology by inducing the formation of large lamellipodia and high numbers of filopodia and defining the strength of cell-cell adhesion by controlling the polarized presentation of vascular endothelial (VE)-cadherin at the cell surface ^45^. In the stalk (follower cell), on the other hand, active Notch signalling interacts with the Wnt/PCP pathway to define its polarity and differentiation (lumen formation). In consequence, transcriptional control through Notch lateral inhibition initially sorts out leader cells and then maintains the identity of follower cells. Dynamic identity allocation, that is concomitant with movement, can also be achieved by asymmetric cell division ^46^. To summarize, in this system, tip cells have a highly dynamic actin cytoskeleton with a large protrusion and are mechanically coupled via cadherins and actomyosin to their direct followers ^47^. Functionally, epithelial/endothelial fingers or strands resemble a bike with multiple riders ( Figure 1A). All riders are interconnected, they all contribute physically but the front rider sets the directionality of the course.

Border cells in *Drosophila* ovaries form a small cluster of about eight cells in total, organized around a core of two immobile cells named polar cells ^48^. They migrate through the surrounding nurse cells towards the oocyte in two distinct ways. An almost linear migration (the running mode) dominates during the earliest phase towards the oocyte. Running is characterized by protrusive activity essentially restricted to the front cell and oriented towards the nurse cells facing it ^49^. The late phase of displacement, near the oocyte, is dominated by the rotating mode of migration. During rotation, all cells display protrusive activity, protrusions are even detected in between border cells and some neighbour exchange can take place ^49^. During the running part of the migration, the cluster has one front cell at a time. The role of leader is taken by the cell that better responds to the external gradient of molecules secreted by the oocyte, including PVF (platelet-derived/vascular endothelium-derived growth factor homologue) and epidermal growth factor (EGF) ligands ^48, 50^. The cell with the highest receptor tyrosine kinase signalling levels in turn generates higher Rac1 levels, resulting in the formation of a stronger protrusion, which allows this cell to take the leading role. As in sheets, the mechanical coupling of the cells via cadherin junction within the cluster prevents other cells from forming protrusions ^51– 55^. Impairing this physical coupling induces the formation of protrusions in all cells and loss of directional migration. This is in agreement with the observation that during the rotating phase all cells display high protrusive activity ^49^. Furthermore, mechanical coupling is such that experimental activation of Rac1 in a cell different from the leader ^56^ inhibits protrusion formation elsewhere in the group and migration is redirected to a new location. In summary, in border cells, during the linear migration phase, a leader emerges in response to an environmental chemoattractant and leader-follower roles are maintained via mechanical coupling preventing excessive protrusive activity in non-front cells. All outer, migratory cells of the group contribute motile force, but the lead cell likely contributes more than followers ^13^. Occasionally, a follower cell takes over the lead position and this suggests that there is nothing unique in the identity of the lead cell. The reasons behind this turnover remain unknown. Although desensitization by endocytosis of the guidance receptor may explain this behaviour, cell turnover has been proposed as a mechanism to prevent desensitization in large groups of chemotactic immune cells ^57^. Functionally, the border cell cluster can be compared to a sedan chair ( Figure 1B). Several active persons carry passive travellers. All carriers are mechanically coupled to each other and to their passive load, but the front carriers set the direction of the course.

Mammary glands develop by progressive branching of an initial epithelium ^58^. Each branch has a terminal end bud migrating into the stroma. End buds are formed of non-protrusive front cells, called cap cells ^59^, and ensheathing terminal end bud body cells that collectively migrate without direct physical contact with the local environment ^59^. Branching is thought to depend on remodelling of the extracellular matrix by MMPs expressed by end bud cells and stromal cells. However, there is no *in vivo* evidence that mammary epithelial cells degrade their basement membranes. Furthermore, recent *in vivo* data indicate that MMP14 and 15 found in the terminal end bud during branching are required for non-catalytic purposes (see 60 for further discussion). Therefore, in this system, front cells are not pathfinding, they do not actively remodel the matrix *in vivo* and the bulk of the traction force is coming from motile end bud body cells that do not occupy the leading edge position.

Ganglia of the cranial nerves VII, IX and X are formed by neural crest and epibranchial placodal cells ^61^. These placodes are epithelial cells located laterally to the neural plate at early stages of development ^62^. Epibranchial placode precursors collectively migrate ventrally and progressively split into distinct subgroups distributed along the antero-posterior axis. Interestingly, placodal cells are motile but lack directionality on their own. They produce a chemokine CXCL12 that attracts nearby, dorsally located, neural crest cells. When a physical contact occurs, N-cadherin–dependent contact inhibition of locomotion promotes the collapse of cell protrusion on both cell populations ^63^. However, neural crest cells are more motile and systematically fill the gap. This difference in motility biases the direction of movement such that neural crest cells progressively repel placodes to advance ventrally. The two populations migrate in a coordinated manner with placodes at the front and neural crest cells at the back displaying a chase-and-run (attraction-repulsion) behaviour ^63^. Contrary to the CAF-cancer cell situation, in this case neural crest cells are the cells that set the directionality to the group and the directional migration of placodal cells is not initiated by cells present at the front of the placode population. It is the transient, repeated, physical contacts between neural crest cells and placodes, occurring at the back of the placode population, that consistently inhibit the formation of lasting protrusions by back placodal cells. This mechanism establishes a front-rear polarity across the placodal group and sustains their ventral-ward directional movement. At the single level, the importance of the back (or rear) for the emergence and stabilization of polarity is well known and has been discussed elsewhere in the context of collective cell migration ^4, 64^. The heterotypic neural crest/placode relationship works similarly to a sheepdog (neural crest) and cattle (placodes) situation ( Figure 1C).

Overall, data on epithelial cells show that directional collective migration can occur in groups where (i) traction forces and matrix remodelling capabilities are shared among migratory cells even when a morphologically distinct tip cell is present (epithelial fingers, vessels sprouting, border cells), (ii) front cells do not display extensive protrusive or matrix remodelling capabilities (mammary gland), and (iii) specific events take place at the rear rather than at the front of the population (placodes).

## Mesenchymal models of collective cell migration

The main models for the analysis of collective migration in mesenchymal cells are the neural crest and the lateral line primordium populations. Neural crest cells are a highly migratory population that emerges from the dorsal neuroepithelium and colonizes the entire developing embryo ^65^.

Cephalic neural crest cells from *Xenopus* and fish embryos polarize according to their contact with other cells and the cell-free space through contact inhibition of locomotion (CIL), which is mediated by homophilic cadherin contacts ^66, 67^. In an unbiased environment, CIL promotes the radial dispersion of cells. This is simply because cells facing the cell-free interface do not experience CIL all around their cell membrane. Cells polarize accordingly, forming a protrusion towards the cell-free space and move away from the main group. *In vivo*, this mechanism is reinforced by confinement ^68^ and the chemoattractant CXCL12 that positively biases the protrusion lifetime ^69^. *In vitro*, cells at the front of the group at any given time present larger and more stable protrusion than the rest of the cells. Yet their actual impact on the direction of followers has not been established. Indeed, the combination of cell cooperation via adhesion ^69^, paracrine signalling ^70^ and confinement ^68^ is sufficient to promote directional movement in the absence of chemotaxis ^9, 68^.

Experiments in chick embryos combining transcriptomic analysis and computational modelling support the importance of cell-cell contact for neural crest migration ^71– 73^ but bring into play different roles for cells at distinct positions of the group ^74– 77^. A small number of specialized trailblazer cells at the migration front, presenting a stable and characteristic transcriptomic signature, would be the only cells that respond to a gradient of the chemoattractant VEGF. Accordingly, trailblazers direct the movement of follower cells directly in contact with them. These followers in turn contact cells further back, forming chains of directionally migrating cells. The gradient of VEGF would be sculpted by the neural crest population as a consequence of the different response to VEGF of front and back cells. While trailblazers could bind and respond to VEGF, follower cells can only bind and consume the factor, acting as a sink ^78^. While this is an interesting proposition, the existence of a VEGF gradient remains speculative. In addition, the importance of the transcriptomic differences observed for the migratory behaviour of cephalic chick neural crest has not been functionally tested.

A different analysis using *in vivo* tracking of cephalic neural crest in chick and fish embryos revealed that the relative position of cells within the group is not stable during migration ^79^. Cells intermingle every time they move the equivalent of one cell diameter (about every 50 minutes), and only 5% of the cells retain the front position throughout migration. Furthermore, the migration of the group is not altered by the total ablation of front cells. Together, these observations show that all cells in the group have the capability to imbue directionality and that cells occupy the front positions only transiently. One possibility for such quick adaptation would be via the selective presentation of CXCR4 at the cell surface. CXCR4, the main receptor of CXCL12, could localize at the cell membrane in a cell-cell adhesion–dependent manner downstream of CIL. The activity of the exocyst complex is influenced by cadherin junctions ^80^, and cytoplasmic pools of CXCR4 are driven to the plasma membrane by this pathway in human cancer cells ^81^. Therefore, as soon as a cell reaches the migration front and acquires a free edge, differential endocytosis would enrich the guidance receptor CXCR4 at the front of the cell. This mechanism would explain the seemingly immediate acquisition of leader traits as it does not require transcriptional or translational delays. In such a case, the characteristic trailblazer transcriptomic signature would be the consequence, and not the cause, of leading edge incorporation.

Alternatively, cells at the front of the cephalic neural crest population may not represent a subgroup of specialized cells. Leader/trailblazer identity would not exist as such. Interestingly, CXCR4 mRNA is detected in the whole population in a salt-and-pepper manner in chick ^82, 83^, fish ^84^, mouse ^82^ and *Xenopus* ^69^ embryos. Thus, CXCL12-responding cells are expected to be found throughout the migrating group. *In silico* data, previously discussed, indicate that chemotaxing cells within a migrating group can influence the directionality of their neighbours if a functional mechanical connection exists (that is, CIL for neural crest) ^10^. Thus, CXCL12 signalling could very well bias migration by acting only on CXCR4-positive cells that are scattered within the population. In this case, there would be no need for a specialized group of front cells or a fancy (and speculative) contact-dependent endocytosis of CXCR4. Similarly to the widespread expression of CXCR4, numerous proteolytic enzymes from the MMP and ADAM families (that is, MMP2/8/9/14/15/17 and ADAM9/10/13/19) are expressed throughout the cephalic neural crest population in chick ^85– 88^, mouse ^89^, fish ^90– 92^ and *Xenopus* ^93– 98^. This strongly suggests that proteolytic capabilities are shared among cephalic neural crest cells and might further explain why specific ablation of front cells did not impair migration.

In the trunk of fish embryos, neural crest cells migrate as a single file of loosely interacting cells. Leader cells at the front of the group are a permanent population and the only cells capable of defining the directionality of the group—traits that define them as leaders. Moreover, leader and follower identities are established before initiation of migration and are not exchangeable during migration ^79^. The molecular pathways controlling identity specification and maintenance of trunk fish neural crest leader cells remain to be unveiled, but it is tempting to make analogies with epithelial sheets and sprouting angiogenesis where the Notch pathway plays a pivotal role.

The posterior lateral line primordium is a 150-micrometre-long heterogeneous migratory population composed in its two front thirds of mesenchymal cells while its rear third is epithelial. This structure migrates along the antero-posterior axis during late phases of fish and amphibian development to deposit mechanosensitive organs, the neuromasts ^99^. The front mesenchymal cells are motile and display polarized and highly protrusive activity at the front of each cell. The rear cells are deposited as epithelial rosettes and are barely motile. The front and rear subpopulations are generated via Wnt/FGF-dependent communication between cells at the onset of migration ^99^. Directionality of migration in this system is set by a gradient of the chemokine CXCL12 ^100^. It has been shown that CXCL12 is homogeneously expressed along the antero-posterior axis and that the formation of a gradient results from different expression of CXCL12 receptors in front and rear populations. CXCR4 is found in all cells, whereas CXCR7 expression is restricted to epithelial rear cells ^100– 103^. CXCR4 is responsible for the chemotactic response to CXCL12, a capacity of all cells in the population ^101^. CXCR7, on the other hand, acts as a decoy receptor that internalizes CXCL12 at the rear of the group ^104^. In this way, the differential localization and activity of these receptors are responsible for a self-generated local gradient of CXCL12 that spans the whole migratory group and creates the directional bias ^101, 102^. Removing rear cells by laser ablation ^105^, or inhibiting CXCR7 expression ^103^, is sufficient to block directional migration even if protrusive activity and CXCR4 expression are not affected in front cells. Interestingly, CXCR7 expression is restricted to rear cells in a CXCR4-dependent manner, such that CXCR4 knockdown leads to widespread expression of CXCR7 through the primordium, lack of gradient formation and misguided migration ^103^. Similar to the migration of cranial placodes, here it is a specific event (local epithelialization and restricted CXCR7 expression) occurring at the back of the migrating group that is responsible for steering the whole population in the right direction. The lateral line primordium functions as a horse-drawn carriage ( Figure 1D) in which a non-mobile rear end sets the course of an otherwise non-directional mobile group.

## Conclusions

The current nomenclature of leader and follower cells ties the position of a cell to its function. Moreover, it implies that front cells represent a particular subgroup of cells that guide the population by exploring the local environment, finding the path, remodelling the matrix and exerting significant traction force. In the meantime, followers are deemed passive cells mostly defined by their inability to generate their own migratory path.

However, the discussed experimental data and *in silico* simulations show that (i) the proposed characteristics of leader cells (pathfinding, traction force, matrix remodelling) are not systematically associated with front cells, (ii) directional collective migration can emerge from homogenous cell populations subjected to external information (gradients, confinement), and (iii) when specialized cells are present these are not necessarily organized as a specific subgroup or positioned at the leading edge. In this context, we think that the leader cell terminology should be simplified and restricted to a specific subset of cases. Leaders are defined as front cells that imbue directionality to the entire group. They may do so in various non-exclusive ways such as displaying an intense protrusive activity, specifically expressing a guidance receptor. The particular mechanism may differ depending on the population studied and the local environment in which migration takes place.

In order to have a term that could apply to all situations, we propose to name the cells that set the overall directionality of a collectively migrating group: steering cells. To steer means “to guide in a particular direction or manner”; we think it is an appropriately ample term that does not imply how the guidance is achieved and that does not associate it with a particular location. That is a general term based solely on a cell’s function that does not overlap and therefore can be used in combination with purely topological terms such as front and rear (or back). Steering cells may be at the front or not. They may be motile or not. Therefore, when studying what controls directionality, one should (i) experimentally test whether the population is homogenous (Are all cells required for directionality? Are some subgroups required and others dispensable?), (ii) explore the mechanisms at play (external gradient, paracrine communication, physical contact, and confinement) and (iii) define which cells respond to what signals.

This concept of steering cells does not preclude the possibility of complex systems where some cells scattered throughout the population might chemotax while a subset of front cells could exhibit proteolytic activities, for instance. There may be several categories of steering cells within a collective.
